# Pyrotinib in the treatment of human epidermal growth factor receptor 2-positive metastatic breast cancer: A case report

**DOI:** 10.1097/MD.0000000000020809

**Published:** 2020-06-19

**Authors:** Jiali Dai, Yuetong Chen, Cuiju Tang, Xiaowei Wei, Yang Gong, Jingsun Wei, Dongying Gu, Jinfei Chen

**Affiliations:** a Department of Oncology, Nanjing First Hospital, Nanjing Medical University; b Cancer Center, Taikang Xianlin Drum Tower Hospital, Nanjing University; c Collaborative Innovation Center for Cancer Personalized Medicine, Nanjing Medical University, Nanjing, China.

**Keywords:** human epidermal growth factor receptor 2-positive, metastatic breast cancer, pyrotinib

## Abstract

**Rationale:**

Pyrotinib is a novel dual pan-ErbB receptor tyrosine kinase inhibitor, approved for the treatment of human epidermal growth factor receptor 2 (HER2)-positive metastatic breast cancer (MBC). However, there was still limited information regarding specific effect of pyrotinib on HER2-positive MBC patients with phosphoinositol-3 kinase mutation.

**Patient concerns:**

A 63-year-old woman accidentally discovered a left breast lesion. The breast cancer was diagnosed by biopsy of breast lesion and postoperative pathological examination in March, 2017. The patient was presented with HER2-positive (3+), invasive carcinoma of the left breast with lymph nodes and lung nodules metastasis, and the clinical stage was T4N2M1. However, the lesion continued to aggressive disease progression with the treatment of trastuzumab plus multiple chemotherapy regimens and traditional Chinese medicine.

**Diagnoses:**

The woman was diagnosed with invasive carcinoma of the left breast and lymph nodes and lung nodules metastasis.

**Interventions:**

The patient received 6 cycles of pyrotinib in combination with capecitabine regularly.

**Outcomes:**

Progression free survival was more than 6 months, and the patient's efficacy evaluation was partial remission.

**Lessons:**

Our clinical observations demonstrated that pyrotinib may be an effective treatment for patients with HER2-positive MBC.

## 1 Introduction

Breast cancer is one of the most frequent diagnosed cancers and the first leading cause of cancer death in females worldwide.^[1]^ Over the decades, the incidence of breast cancer was increased significantly, and the age of onset has gradually become younger, which is a serious threat to women's physical and mental health.^[1]^ Most patients are already at the advanced stage of diagnosis and lose chance to receive curative (R0) surgical resection. With the deepening of research on the genes and molecular mechanisms of malignant tumors, targeted therapy has gradually become an effective treatment option.^[2]^ Targeted therapy is widely used in the treatment of metastatic breast cancer (MBC), combined with chemotherapy, radiotherapy, and endocrine therapy. Targeted drug can reduce cancer symptoms, improve quality of life, and prolong survival time.^[3]^ Approximately 15% to 20% of patients with breast cancer have overexpression of human epidermal growth factor receptor 2 (HER2).^[4]^ HER2-positive breast cancer is a more aggressive phenotype, that is prone to recurrence and has poor prognosis.^[5]^ With the development of anti-HER2 targeted therapy,^[6]^ there was a substantial improvement in survival of patients with HER-2 positive MBC.^[2]^ HER2 is a tyrosine kinase receptor,^[7]^ and extracellular HER-2 protein binding domain binds to the ligand, thereby affecting the phosphorylation of the intracellular kinase domain and the formation of the corresponding dimer.^[8]^ The bindings can activate Ras/Raf/mitogen-activated protein kinase (MAPK), phosphatidylinositol-3-kinase/ protein kinase B (P13K/Akt), signal transducer and activator of transcription (STAT), and phospholipase C (PLC) intracellular signaling pathways, thereby inhibiting apoptosis and promoting cell proliferation.^[7]^ Moreover, they can increase the invasiveness of tumor cells and promote the formation of tumor blood vessels, resulting in diverse antitumor biological effects.^[8]^ Trastuzumab is the first anti-HER2 monoclonal antibody, reducing both the recurrence rate and mortality, promoting the development of other anti-HER2 drugs.^[2]^ Trastuzumab can bind to the extracellular domain of the HER2 receptor and interfere with the formation of heterodimers, thereby inhibiting tumor formation.^[9]^ Many clinical studies indicated trastuzumab had a significant improvement in disease-free and overall survival with HER2-positive breast cancers.^[9]^ Regretfully, approximately 15% of breast cancer patients receiving trastuzumab develop drug resistance or even recurrence during the treatment.^[10]^ Therefore, we need to explore additional agents to overcome resistance and improve patient outcomes.^[11]^

Pyrotinib is a novel, irreversible dual pan-ErbB receptor tyrosine kinase inhibitor developed by Shanghai Hengrui Pharmaceutical (a subsidiary of Jiangsu Hengrui Medicine, Minhang, Shanghai, China) for the treatment of HER2-positive advanced solid tumors, particularly breast cancer.^[5]^ With activity against epidermal growth factor receptor/HER1, HER2, and HER4, pyrotinib can reverse the resistance of trastuzumab and significantly improve the outcome of HER2-positive MBC.^[5]^ Several studies showed pyrotinib had promising antitumor activity in HER2-positive patients with MBC.^[12]^ The phase I/II clinical trial demonstrated that pyrotinib plus capecitabine combination therapy had a promising objective response rate (ORR) and survival rates.^[13,14]^ Based on positive results from the clinical trial, pyrotinib was recently approved in China, and combined with capecitabine for the treatment of HER2-positive, advanced or metastatic breast cancer in patients previously treated with anthracycline or taxane chemotherapy.^[5]^ Here, we reported a case of HER2-positive MBC that was successfully treated with pyrotinib plus capecitabine, when trastuzumab plus multiline chemotherapy regimen was unsuccessful. In addition to regression of the lesion and axilla lymph nodes, the patient was described a significant reduction in lung nodule metastasis.

## 2 Case report

A 63-year-old woman was originally admitted to an outside facility due to left breast lesion in March, 2017. The patient was diagnosed with breast cancer after biopsy of breast lesion and postoperative pathological examination. She was presented with estrogen receptor-negative, progesterone receptor-positive (1+), and HER2-positive (3+), invasive carcinoma of the left breast with lymph nodes and lung nodules metastasis, and the clinical stage was T4N2M1. Moreover, phosphoinositol-3 kinase (PIK3CA) mutation was discovered using “Next-generation” sequencing technology. In the past 2 years, the patient has received targeted therapy and multiple chemotherapy regimens including “epirubicin plus cyclophosphamide,” “trastuzumab plus docetaxel,” and “trastuzumab, docetaxel plus carboplatin.” Although treated with targeted therapy plus multiple chemotherapy regimens, no significant difference in the left breast lesion and the left axilla lymph nodes were found. After 1-year chemotherapy treatment, she refused to receive chemotherapy as maintenance therapy, instead, switched to treatment with traditional Chinese medicine.

**Figure 1:**
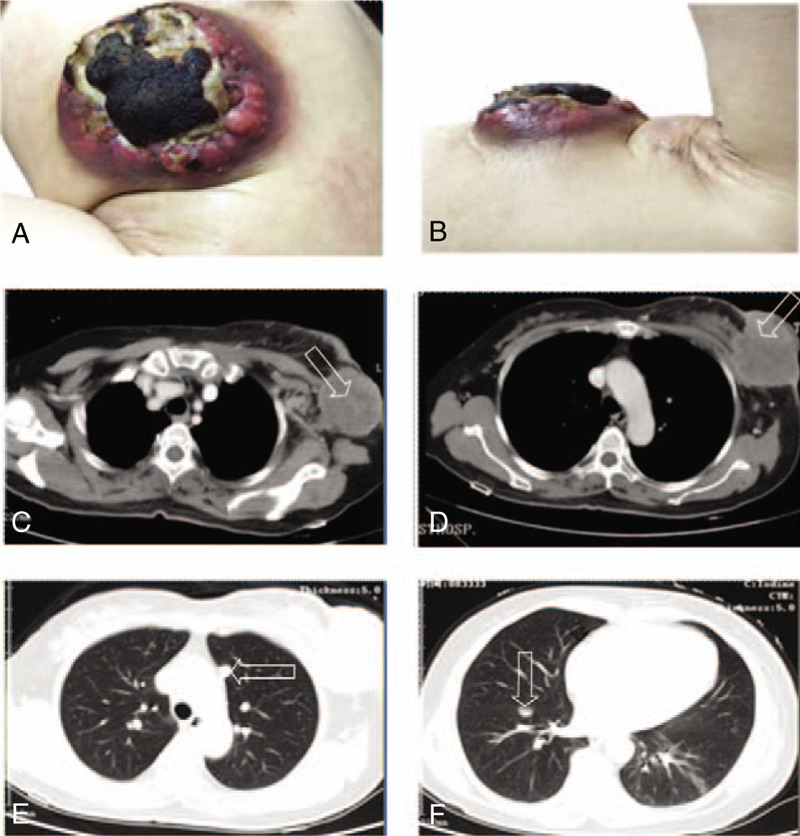
Wound pictures and cross-sectional images before treatment with Pyrotinib. The size of the breast mass was about 10 cm ^∗^ 10 cm ^∗^ 7 cm on October 15, 2018 (A and B). The chest routine scan revealed huge left breast lesion, abnormal left axillary lymph nodes and multiple lung nodules metastatic (C–F). The patients’ response to pyrotinib therapy was consistent with a partial remission.

**Figure 2:**
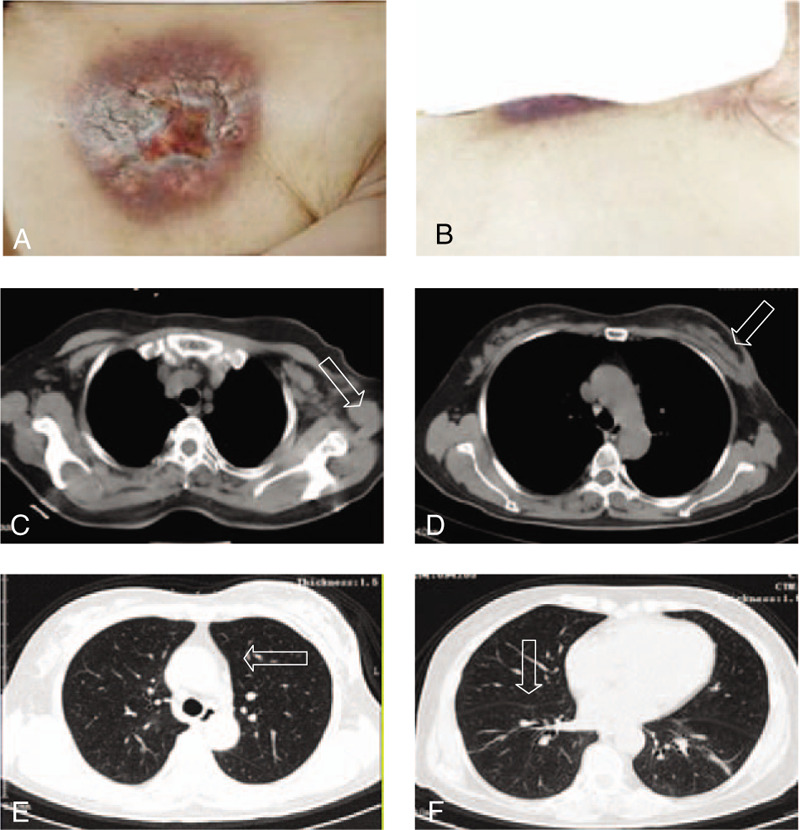
Wound pictures and cross-sectional images after 2 cycles of treatment with Pyrotinib. The lung nodules, breast lesion and axillary lymph nodes obviously shrinked (A–F). The patients’ response to pyrotinib therapy was consistent with a partial remission.

**Figure 3:**
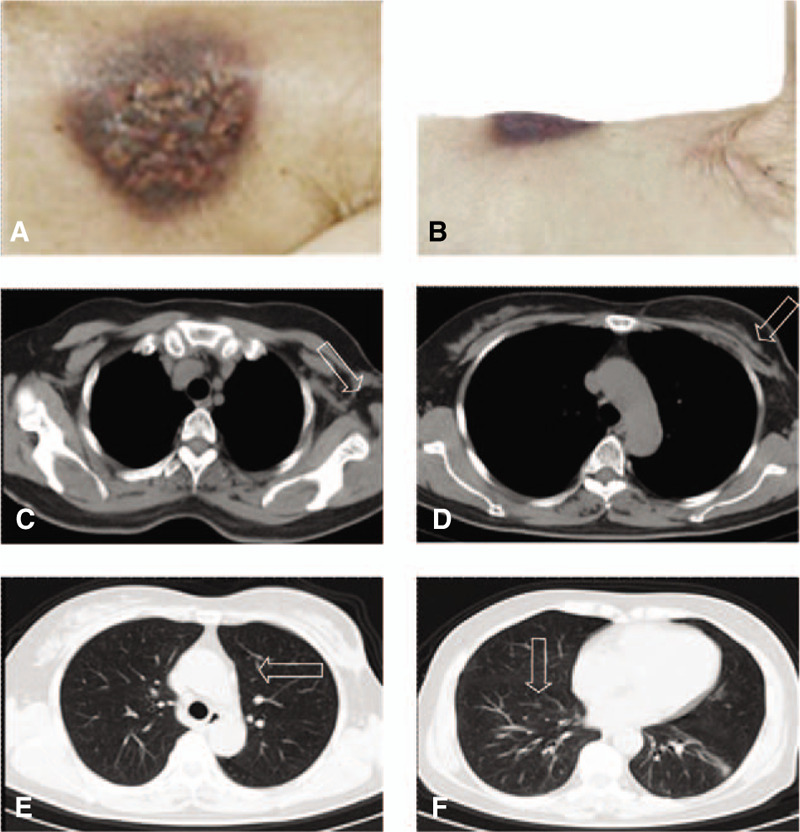
Wound pictures and cross-sectional images after 6 cycles of treatment with Pyrotinib. The lung nodules completely disappeared, breast lesion and axillary lymph nodes obviously shrinked (A to F). The patients’ response to pyrotinib therapy was consistent with a partial remission.

In the following year, the left breast lesion and the left axilla lymph nodes of the woman were enlarged. The woman frequently had low fever and pain with the surface of the left breast lesion festering and bleeding at the same time. By roughly measuring, the size of the breast mass was about 10 cm ∗ 10 cm ∗ 7 cm on October 15, 2018 (Fig. 1A and B). Meanwhile, the chest routine scan revealed huge left breast lesion, abnormal left axillary lymph nodes, and multiple lung nodules metastatic (Fig. 1C–F). The patient had poor curative effect to trastuzumab, therefore, she began receiving pyrotinib (400 mg once daily on days 1–21) and capecitabine (1000 mg/m^2^ twice daily on days 1–14) every 3 weeks. In the following weeks, there was a regression of the breast lesion, axillary lymph nodes, and lung lesions. Figure 2A–F demonstrated wound pictures and cross sectional images after approximately 2 cycles of the treatment with pyrotinib and capecitabine. According to RECIST 1.1 criteria, the patients’ response to pyrotinib therapy was consistent with a partial remission. She remained on pyrotinib plus capecitabine regimen for 6 cycles with a continued partial response; the lung nodules completely disappeared, breast lesion and axillary lymph nodes obviously shrinked (Fig. 3A–F).

According to Common Terminology Criteria for Adverse Events, the patient had manageable adverse events such as grade 2 diarrhea, grade 1 hand-foot syndrome, and grade 2 leucopenia. Diarrhea generally occurred during the first 4 days of the treatment, lasted for 2 to 3 days. She was managed with symptomatic treatment (eg, loperamide, montmorillonite, compound eosinophil-lactobacillus), without suspending or decreasing the dosage of pyrotinib and capecitabine. Diarrhea was gradually relieved with the patient's treatment cycle increasing. Other adverse events such as hand-foot syndrome and leukopenia were controlled by symptomatic treatment (eg, urea ointment, recombinant human granulocyte colony-stimulating factor).

She was treated with pyrotinib plus capecitabine continuously. Progression free survival (PFS) was more than 6 months, and the patient's efficacy was assessed as partial remission.

## 3 Discussion

The case above indicated that pyrotinib can be an effective treatment for a patient with HER2-positive MBC. HER2-positive breast cancer accounts for approximately 15% to 20%, and the particular subtype of breast cancer is more aggressive.^[5]^ With the development of HER2-targeted therapy, the prognosis and clinical efficacy of breast cancer patients have been greatly improved.^[15]^ Trastuzumab is a targeted drug with substantial anti-tumor efficacy and is generally well tolerated for the treatment of advanced HER2-positive breast cancer.^[15]^ Some patients exhibit primary resistance to trastuzumab due to mutation in PIK3CA.^[16]^ The patient who received trastuzumab in the first cycle did not achieve significant efficacy and may have primary resistance to trastuzumab. However, the majority of patients eventually have resistance to trastuzumab after long-term treatment.^[15]^ Mutations in PIK3CA was reported to correlate with the outcome of anti-HER2 treatment.^[12]^ Dave et al's study showed activating mutation in PIK3CA conferred resistance to the trastuzumab.^[17]^ PIK3CA mutations were found in the woman, and were associated with worse outcomes after trastuzumab treatment.^[17]^

Pyrotinib is a novel tyrosine kinase inhibitor with multiple targets that has a more pronounced clinical efficacy on inhibiting tumor growth.^[12]^ Pyrotinib can covalently bind to the ATP binding site of the intracellular kinase domain of HER1, HER2, and HER4, preventing the formation of HER family homologous/heterodimer, inhibiting auto phosphorylation, and blocking the activation of downstream signaling pathways.^[18]^ Several studies showed pyrotinib was adjuvant treatment for patients with HER2-positive MBC.^[12]^ A phase I clinical trial conducted by Li et al demonstrated that pyrotinib plus capecitabine combination therapy had a promising ORR of 78.6% and a TTP of 22.1 months.^[13]^ Most of all, a pivotal phase II trial compared pyrotinib plus capecitabine combination therapy with lapatinib plus capecitabine combination therapy in patients with HER2-positive MBC.^[14]^ Moreover, there was a significant difference in median PFS between pyrotinib arm and lapatinib arm (18.1 vs 7.0 months, *P* < .01).^[14]^ Pyrotinib group was associated with a significant ORR versus lapatinib group (78.5% vs 57.1%, *P* = .01).^[14]^ In addition, the subgroup analysis showed that pyrotinib group significantly prolonged PFS, regardless of the patients who received trastuzumab previously for advanced disease.^[14]^

Besides that, our findings suggested pyrotinib was a viable alternative to the treatment of HER2-positive MBC, even if the lesion is resistant to trastuzumab and chemotherapy. Previous studies indicated that the mechanism of trastuzumab resistance is related to PIK3CA mutations.^[19]^ The patient was tested for PIK3CA, but remained sensitive to pyrotinib-containing treatments, and there is no limitation with treatment of pyrotinib. Therefore, these two therapeutic agents may have different cellular mechanisms on cell survival and apoptosis. The study demonstrated that the mechanism of HER2 drug resistance may be related not only to PIK3CA mutations. The mutations of PIK3CA can predict resistance to trastuzumab, but does not predict resistance to pyrotinib. However, mechanism for pyrotinib resistance was not well established,^[20]^ and such clinical trials are currently underway.^[17]^ There is no clinical study to compare pyrotinib plus capecitabine versus capecitabine monotherapy or pyrotinib monotherapy. Although the woman was successfully treated with a combination of pyrotinib and capecitabine, we were unable to definitively rule out the complimentary action between the pyrotinib and capecitabine. We need further clinical trials to compare pyrotinib plus capecitabine versus capecitabine monotherapy or pyrotinib monotherapy in the future research and have obtained more comprehensive conclusion.

Previous studies suggested that overexpression of HER2 is a frequent molecular abnormality in primary breast cancer and primary gastric cancer.^[21]^ We are aware of that more studies are required to better understand how pyrotinib acts on HER2-positive breast cancer and HER2-positive gastric cancer.^[22]^ Moreover, a randomized clinical trial would be important to demonstrate efficacy in HER2-positive gastric cancer. Additional studies are needed to elucidate pyrotinib's exact mechanism of action, and we will begin to analyze of other HER2-positive solid tumors in the near future.

In this case, we demonstrated that pyrotinib seems to provide an effective and easily tolerated therapy of HER2-positive MBC, and can lead to a significant improvement in disease burden, the quality of life, and survival time.

## Author contributions

**Conceptualization:** Jiali Dai, Dongying Gu, Jinfei Chen.

**Data curation:** Jiali Dai, Yuetong Chen, Jingsun Wei.

**Formal analysis:** Jiali Dai, Yuetong Chen, Xiaowei Wei, Yang Gong.

**Funding acquisition:** Dongying Gu, Jinfei Chen.

**Investigation:** Dongying Gu.

**Methodology:** Jiali Dai, Cuiju Tang, Xiaowei Wei, Jingsun Wei.

**Project administration:** Jiali Dai.

**Resources:** Jiali Dai.

**Software:** Jiali Dai, Yuetong Chen, Cuiju Tang, Xiaowei Wei, Yang Gong, Dongying Gu.

**Supervision:** Dongying Gu, Jinfei Chen.

**Validation:** Dongying Gu, Jinfei Chen.

**Visualization:** Jiali Dai, Yuetong Chen, Cuiju Tang, Yang Gong, Dongying Gu.

**Writing – original draft:** Jiali Dai, Dongying Gu.

**Writing – review & editing:** Jiali Dai, Dongying Gu, Jinfei Chen.
